# Validation of point-of-care tests used at in-home assessments among older adults in primary care

**DOI:** 10.1080/02813432.2024.2426162

**Published:** 2024-11-11

**Authors:** Siri Aas Smedemark, Ditte Beck Jepsen, Karen Andersen-Ranberg, Mads Nybo

**Affiliations:** a Geriatric Research Unit, Department of Geriatric Medicine, Odense University Hospital, Odense, Denmark; b Department of Clinical Research, University of Southern Denmark, Odense, Denmark; c Department of Clinical Biochemistry, Odense University Hospital, Odense, Denmark

**Keywords:** Point-of-care test, older adults, in-home assessments, validation, biochemical analyses

## Abstract

**Background:**

Diagnosing acute disease in older adults is challenged by vague and atypical symptoms. Point-of-care tests (POCTs) at home may improve diagnostics and clinical decision-making. We compared various POCT devices to routine testing in acutely ill older adults to assess their clinical reliability.

**Methods:**

We enrolled participants aged 65+ years requiring acute in-home assessment with signs of acute conditions. Venous and capillary blood samples were collected and analysed on-site using POCT, while identical samples were transported and analysed in a routine laboratory. Agreement between POCT and laboratory testing was assessed using scatter plots with linear regression, Pearson’s correlation coefficient (PCC), limits of agreement, and Bland-Altman plots. Misclassification rates were calculated based on clinically meaningful cut-offs to assess POCT’s clinical reliability.

**Results:**

We included 100 participants with a mean age of 81.6 (±8.4 SD) years. Strong correlation was found between POCT and routine measurements (PCC: 0.76–0.94 for capillary samples and 0.85–0.98 for venous samples). Venous samples showed higher PCC than capillary, except for neutrophils (0.93 for capillary, 0.89 for venous). Misclassification occurred in capillary samples for haemoglobin (10/62) and total WBC (6/50), while in venous samples, misclassification was observed for haemoglobin (4/54), total WBC (4/50), K^+^ (5/47), urea (5/47), and creatinine (3/42). No misclassification was observed for Na^+^.

**Conclusion:**

POCT provides acceptable, clinically reliable measurements in acutely ill older adults, potentially enhancing diagnostics and treatments during in-home assessment. Venous blood testing is preferable due to a lower misclassification rate, but capillary blood remains a pragmatic alternative, despite higher variation and inaccuracy.

## Introduction

Point-of-care tests (POCTs) offer a convenient alternative to traditional laboratory testing by providing rapid and accessible diagnostic information directly at the patient’s side. These tests have shown promising potential to support clinical decision-making, potentially reducing hospital stays, improving treatment adherence, and lowering complications [1]. With their compact design, POCT devices have also found application in prehospital care settings [2]. Common POCTs for C-reactive protein (CRP), haemoglobin, international normalised ratio (INR), and blood glucose are widely utilized by primary care physicians, demonstrating their potential to guide diagnostics and treatment decisions in a primary care setting [3, 4]. Recent advances in POCTs technology have introduced new tests, such as white blood cell (WBC) differential count, with devices like the HemoCue WBC DIFF® demonstrating reliability in providing accurate counts in leukopenic samples [5].

Timely diagnosis and treatment decisions in primary healthcare settings have gained considerable attention with the aim of preventing acute hospital admissions [6], especially amid an increasing number of older adults and a simultaneous decline in hospital beds and healthcare professionals in both primary and secondary health care. This imbalance between demand and resources poses a challenge to health care sectors in high- and middle-income countries [6], necessitating innovative solutions to optimize care pathways for older adults. The use of advanced POCT in the older patients’ homes may aid early diagnostics by either excluding a disease or identifying patients, who would benefit the most from further referrals or treatment initiation [7]. Over the last few of decades, the use of POCT in patients’ own home has been explored; however, their performance in older adults suspected of acute conditions remains understudied. Such investigations are imperative since POCT measurements must be used in conjunction with clinical assessments to diagnose dehydration, anaemia, and infections, which are highly relevant conditions in a cohort of older adults suspected of acute disease [8].

To promote the implementation of advanced POCTs during in-home assessments, it is important to determine whether POCTs are as clinically reliable as routine laboratory testing in a cohort of older adults suspected of acute disease. Our study aimed to validate POCTs, including iSTAT®, Hemocue WBF diff®, and Hemocue Hgb 801®, compared to routine laboratory testing in acutely ill older adults.

## Materials and methods

### Study setting and study participants

This validation study was conducted as part of a pilot and feasibility study carried out between September and November 2021 in Kolding Municipality, Denmark [7], in collaboration with the Acute Community Health Care Service (ACHCS). In Denmark, ACHCS’s were established in 2018 to increase timely clinical decision-making in the primary healthcare sector. They are manned by acute community nurses (ACNs) who conduct in-home assessments with simple point-of-care testing (POCT), including C-reactive protein (CRP) and hemoglobin.

This study enrolled older adults aged 65 years and above who were referred for an acute in-home assessment conducted by an ACN. Referrals for in-home assessments were made by primary care physicians or home care personnel. The in-home assessments were conducted by ACNs accompanied by a medical doctor. To be eligible for participation, individuals had to meet at least one of the following inclusion criteria: fever, dyspnoea, chest pain, fall, or functional decline, defined as either subjective (not able to perform normal daily activities) or objective functional decline (increased need of home care service). Blood samples, which are the focus of analysis and results in this paper, were collected during the in-home assessments.

#### Sample size

This study was part of a pilot and feasibility study [7], where a convenient sample of 100–150 participants was chosen to investigate feasibility and potential clinical impact. Therefore, no sample size calculation was performed for this specific study.

### Blood sampling

Capillary blood samples were collected by finger prick by the acute community nurses using single-use lancets. The first drop of blood was discarded. Venous blood samples were collected by venous puncture in lithium/heparin and EDTA-vacutainers by the medical doctor.

For POCT, venous and capillary blood samples were analyzed within one minute after the dedicated micro-cuvettes were filled.

For routine analyses, venous blood samples were transported to the laboratory in a climate bag and a climate control cabinet to maintain a stable temperature of 20 °C. All venous blood samples for routine testing were delivered within two hours to the Department of Biochemistry and Immunology, Hospital Lillebaelt, Kolding. The analyses included sodium (Na^+^), potassium (K^+^), creatinine (Crea), and urea and were analysed on a Cobas 8000 C® (Roche Diagnostics, Mannheim, Germany), while haemoglobin and WBC differential count were analysed using Sysmex XN-9000® (Sysmex, Japan).

### POCT analyses/biochemical analysis

The POCT instrument Hemocue WBF-diff® (HemoCue AB, Ängelholm, Sweden) was used to analyse WBC differential count using dedicated micro-cuvettes. The POCT instrument Hemocue 801® (HemoCue AB, Ängelholm, Sweden) was used to analyse haemoglobin levels using dedicated micro-cuvettes. The POCT instrument iSTAT® (Abbott, Inc., NJ, U.S.A.) was used to analyse Na^+^, K^+^, creatinine, and urea levels using CHEM8+ cassettes. The POCT instruments were calibrated according to the manufacturer’s instructions. Performance characteristics for the POCT instruments are shown in Table 1.

**Table 1.** Overview of biochemical parameters, reference intervals, measured ranges, and clinically relevant cut-offs.

Biochemical parameter (unit)	Measured range	Reference interval	POCT measurement range	CV(%) from laboratory	Clinically relevant cut-offs
Haemoglobin (mmol/L)	– 10.3	♂ 8.3–10.5 ♀ 7.3–9.5	0.62–15.9	<1.0	<7.3
Total WBC (10^9^/L)	1.9–17.6	3.5–8.8	0.3–30.0	<3.0	<3.5 >8.8
Neutrophils (10^9^/L)	0.33–14	2.0–8.8	–	<8.0	<2.0 >8.8
Lymphocytes (10^9^/L)	0.35–4.11	1.0–3.5	–	–	<1.0 >3.5
Na^+^ (mmol/L)	126–144	137–145	100–180	<1	<125
K^+^ (mmol/L)	3.0–5.4	3.5–4.4	2.0–9.0	<2.5	<3.5 >4.6
Urea (mmol/L)	2.1–21.7	♂ 3.5–8.1 ♀ 3.1–7.9	1–50	<3.0	>8.1
Creatinine (µmol/L)	31–260	♂ 60–105 ♀ 45–90	18–1768	<2	>105

Abbreviations: CV; coefficient of variation. POCT; Point-of-care test.

### Statistical analyses

Visual inspection of histograms and the D’Agostino-Pearson’s test were used to test for normal distribution [9]. Scatter plots with linear regression and Pearson’s correlation coefficient were computed to evaluate linear correlations between measurements from POCT and routine laboratory testing. The mean of the difference (SD of difference) between measurements from POCT and routine laboratory testing was calculated, and limits of agreement (LOA) were derived based on a Bland-Altman plot to quantify the agreement between POCT and routine laboratory testing [10].

We aimed to evaluate the clinical reliability of POCT results, by calculating misclassification rates, as similar in other studies [11]. Clinically relevant cut-offs were defined a priori for statistical analysis based on laboratory reference intervals, and for sodium (Na^+^), a value where treatment were deemed relevant [12]. Samples were categorized into predefined groups; under, above, or within the normal reference interval based on these clinically relevant cut-offs. The groups were compared between POCT and routine laboratory test, stratified by capillary and venous blood samples.

Data was entered and stored using RedCap® and analysed using STATA® version 18 software (StataCorp LLC, Texas, USA).

### Ethical considerations

This validation study, which was part of the pilot study but considered a quality assessment project, was registered by the Research & Innovation Organization (RIO), University of Southern Denmark, record of data processing activities (Project identification number: 11.404). The pilot study received ethical approval from the Regional Committees on Health Research Ethics for Southern Denmark (S-20210046). Data was processed and stored in compliance with the EU General Data Protection Regulation (GDPR) and the Danish Data Protection Act. The ethical principles for medical research as stated by the Declaration of Helsinki were applied throughout the study [13]. Written informed consent was obtained from all participants by the principal investigator. Consent procedure, forms, and process followed guidelines and regulation given by the Regional Committees on Health Research Ethics for Southern Denmark and was ethically reviewed by the Committees.

## Results

**Figure 1.**
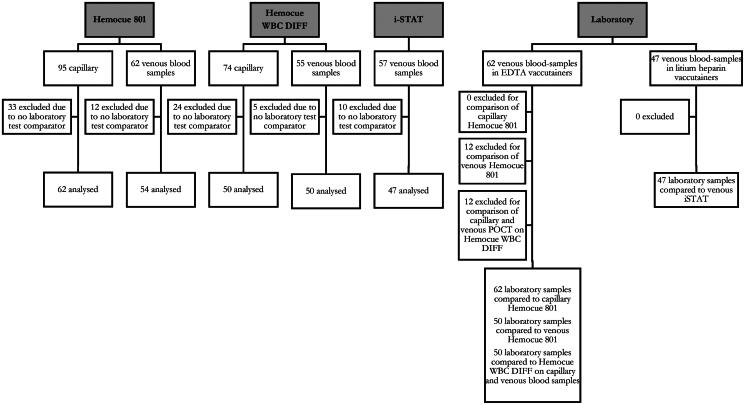
Samples included and excluded divided into routine testing and POCT measurements.

A total of 100 participants were enrolled in the pilot study with a mean age of 81.6 (±8.4 SD) years, of whom 54% were female. Table 2 displays characteristics of the study sample and Figure 1 presents the flowchart detailing the inclusion of samples.

**Table 2.** Characteristics of study sample.

Characteristic	Total study population (*N* = 100)
Age, mean (SD)	81.6 (8.4)
Female, %	54
Place of in-home assessment, %	
Own home	86
Care home	9
Skilled nursing facility	5
Referred by, n	
General practitioner	68
Home care	24
Other*	8
Home care, %	
Receiving home care**	71
Polypharmacy, n	
>5 medications daily	95
Functional level (Barthel 20)	
Mean (SD)	14.9 (5.8)
Clinical Frailty Scale (1–9)	
Very fit, well, managing well (1–3)	26
Vulnerable (4)	19
Mildly frail (5)	18
Moderately frail (6)	15
Severely frail (7)	12
Very severely frail (8)	3
Terminal Ill (9)	7
Median (IQR)	5 (3–6)
Symptoms	
Cough	52
Fever	23
Fatigue	61
Dyspnoea	68
Chest pain	3
Functional decline***	58
Other symptoms^	11
Days ill	
1 to 3 days	52
4 to 7 days	28
> 7 days	20

* Other: acute community nurses, nurses from care homes, palliative nurses.

** Percentage of home care receivers among participants living in own home.

*** Functional decline defined as subjectively not able to conduct daily activities or objectively in need of increased home care.

^^^ Other symptoms: Abdominal pain, muscle pain, fall.

In 95 participants, capillary blood samples were collected and analysed using Hemocue 801®. Five samples were missing due to the acute nature of the patients’ condition, necessitating immediate hospital referrals.

Capillary blood samples were collected and analyzed on Hemocue WBC DIFF® in 74 patients. The primary reason for the missing capillary blood samples on the Hemocue WBC DIFF® was lack of available kits.

Venous blood samples were collected from 89 of the participants. The primary reason for not collecting venous blood samples was technical issues such as difficulties in gaining venous access.

Venous blood samples for routine laboratory testing were collected in EDTA vacutainers for 62 participants and in lithium/heparin vacutainers in 47 participants. The primary reason for missing of 27 and 42 blood samples was attributed to electronic transmission issues within the laboratory’s ordering system. A number of samples were therefore excluded from the analysis (35, 24, and 42 for Hemocue 801, Hemocue WBC DIFF, and i-STAT, respectively) due to the inability to match them with routine laboratory testing.

Table 1 provides information on the measured biochemical parameters, reference intervals, and medians with interquartile range for each specific POCT.

**Figure 2.**
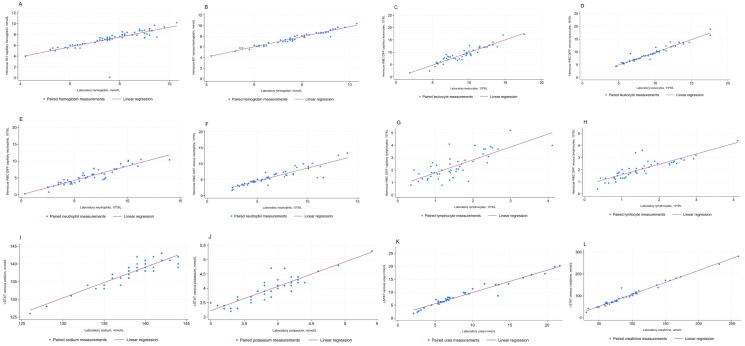
Correlation between point-of-care test measured on capillary samples by Hemocue 801, WBC DIFF system, and i-STAT compared to measurements made by laboratory testing (a): Correlation between hemoglobin levels measured in capillary samples by Hemocue 801 compared to hemoglobin levels on venous samples analyzed by laboratory (b): Correlation between hemoglobin levels measured in venous samples by Hemocue 801 compared to hemoglobin levels on venous samples analyzed by laboratory (c): Correlation between leukocyte levels measured in capillary samples by Hemocue WBC DIFF System compared to leukocyte levels on venous samples analyzed by laboratory (d): Correlation between leukocyte levels measured in venous samples by Hemocue WBC DIFF System compared to leukocyte levels on venous samples analyzed by laboratory (e): Correlation between neutrophil levels measured in capillary samples by Hemocue WBC DIFF System compared to neutrophil levels on venous samples analyzed by laboratory (f): Correlation between neutrophil levels measured in venous samples by Hemocue WBC DIFF System compared to neutrophil levels on venous samples analyzed by laboratory (g): Correlation between lymphocytes levels measured in capillary samples by Hemocue WBC DIFF System compared to lymphocytes levels on venous samples analyzed by laboratory (h): Correlation between lymphocytes levels measured in venous samples by Hemocue WBC DIFF System compared to lymphocytes levels on venous samples analyzed by laboratory (i): Correlation between sodium levels measured in venous samples by i-STAT compared to sodium levels on venous samples analyzed by laboratory (j): Correlation between potassium levels measured in venous samples by i-STAT compared to potassium levels on venous samples analyzed by laboratory (k): Correlation between urea levels measured in venous samples by i-STAT compared to urea levels on venous samples analyzed by laboratory (l): Correlation between creatinine levels measured in venous samples by i-STAT compared to creatinine levels on venous samples analyzed by laboratory.

Figure 2 show the linear correlation between POCT measurements and routine laboratory testing for each specific biochemical parameter.

The Pearson’s correlation coefficients (PCC) are listed in Table 3 and generally indicated a strong correlation between capillary or venous POCT measurements and routine measurements (PCC for capillary samples ranging from 0.76 to 0.94, and PCC for venous samples from 0.85 to 0.98). PCC values were generally higher for venous samples than for capillary, except for neutrophils (PCC 0.93 for capillary and 0.89 for venous samples).

**Table 3.** Point-of-care test measurements compared to routine analyses.

Device	Biochemical parameter	Coefficient of variance (%)	Mean difference (*)		Standard deviation		Pearson correlation coefficient		95% limits of agreement	
			Capillary	Venous	Capillary	Venous	Capillary	Venous	Capillary	Venous
Hemocue 801	Haemoglobin (mmol/L)	2.3	0.45	0.07	1.01	0.27	0.76 *p* value 0.000	0.97 *p* value 0.000	−1.53 to 2.43	−0.47 to 0.60
Hemocue WBF DIFF	Total WBC (10^9^/L)	5.0 2.8 3.1	0.08	0.11	1.07	0.77	0.94 *p* value 0.000	0.97 *p* value 0.000	−2.01 to 2.17	−1.41 to 1.62
	Neutrophils (10^9^/L)	7.3 3.7 4.3	0.93	0.70	0.98	1.27	0.93 *p* value 0.000	0.89 *p* value 0.000	−1 to 2.85	−1.79 to 3.191
	Lymphocytes (10^9^/L)	7.8 4.6 9.5	−0.81	−0.512	0.66	0.42	0.76 *p* value 0.000	0.85 *p* value 0.000	−2.11 to 0.48	−1.33 to 0.30
iSTAT	Na^+^ (mmol/L)	<0.3	–	0.71	–	1.61	–	0.91 *p* value 0.000	–	−2.45 to 3.86
	K^+^ (mmol/L)	<0.4	–	−0.08	–	0.22	–	0.89 *p* value 0.000	–	−0.51 to 0.34
	Urea (mmol/L)	<2.2	–	−0.05	–	1.21	–	0.97 *p* value 0.000	–	−2.42 to 2.31
	Creatinine (µmol/L)	<2.2	–	−6.07	–	9.71	–	0.98 *p* value 0.000	–	−25.10 to 12.96

**Figure 3.**
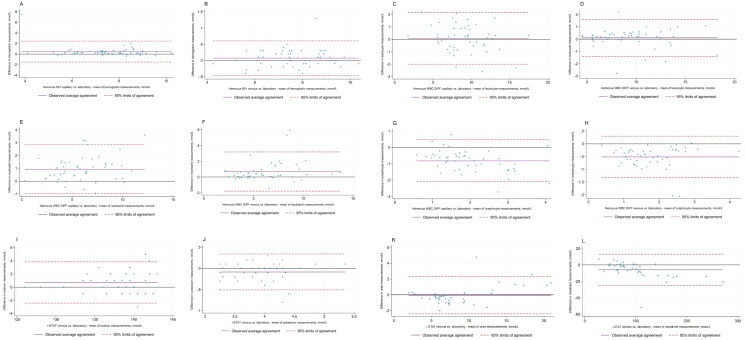
Bland-Altman plots of point-of-care test compared to measurements made by laboratory testing (a): Bland-Altman plot of hemoglobin levels in capillary samples measured by Hemocue 801 compared to hemoglobin levels on venous samples analyzed by laboratory (b): Bland-Altman plot of hemoglobin levels in venous samples measured by Hemocue 801 compared to hemoglobin levels on venous samples analyzed by laboratory (c): Bland-Altman plot of leukocyte levels in capillary samples measured by Hemocue WBC DIFF System compared to leukocyte levels on venous samples analyzed by laboratory (d): Bland-Altman plot of leukocyte levels in venous samples measured by Hemocue WBC DIFF System compared to leukocyte levels on venous samples analyzed by laboratory (e): Bland-Altman plot of neutrophil levels in capillary samples measured by Hemocue WBC DIFF System compared to neutrophil levels on venous samples analyzed by laboratory (f): Bland-Altman plot of neutrophil levels in venous samples measured by Hemocue WBC DIFF System compared to neutrophil levels on venous samples analyzed by laboratory (g): Bland-Altman plot of lymphocytes levels in capillary samples measured by Hemocue WBC DIFF System compared to lymphocytes levels on venous samples analyzed by laboratory (h): Bland-Altman plot of lymphocytes levels in venous samples measured by Hemocue WBC DIFF System compared to lymphocytes levels on venous samples analyzed by laboratory (i): Bland-Altman plot of sodium levels in venous samples measured by i-STAT compared to sodium levels on venous samples analyzed by laboratory (j): Bland-Altman plot of potassium levels in venous samples measured by i-STAT compared to potassium levels on venous samples analyzed by laboratory (k): Bland-Altman plot of urea levels in venous samples measured by i-STAT compared to urea levels on venous samples analyzed by laboratory (l): Bland-Altman plot of creatinine levels in venous samples measured by i-STAT compared to creatinine levels on venous samples analyzed by laboratory.

Figure 3 displays Bland-Altman plots comparing POCT to measurements from routine laboratory testing. These plots reveal no clear systematic bias, although an increase in outliers is noticeable in the higher measurements of sodium, suggesting a possible increase in bias at higher sodium concentrations.

The evaluation of misclassification is presented in Tables 4 and 5. Using routine laboratory testing as the gold standard, misclassification when employing clinically relevant cut-offs on capillary blood testing occurred in 10 out of 62 for haemoglobin, 6 out of 50 for total WBC, 5 out of 50 for neutrophils, and 20 out of 47 for lymphocytes (Table 4).

**Table 4.** Point-of-care test results on capillary blood samples compared to routine analyses – clinically relevant cut-offs.

			Measurements outside the clinically relevant cut-off				
Device	Biochemical parameter	Sample size	Laboratory		Point-of-care test capillary blood samples		
					Lower limit	Normal	Upper limit
Hemocue 801	Hb, mmol/L	62	Lower limit (<7.3)	20	19	1	–
			Normal (7.3-XX)	42	9	33	–
Hemocue WBF DIFF	Total WBC (10^9^/L)	50	Lower limit (<3.5)	1	1	0	0
			Normal (XX)	21	1	19	1
			Upper limit (>8.8)	28	0	4	24
	Neutrophils (10^9^/L)	50	Lower limit (<2.0)	1	1	0	0
			Normal (2.0–8.8)	39	1	38	0
			Upper limit (>8.8)	10	0	4	6
	Lymphocytes (10^9^/L)	47	Lower limit (<1.0)	12	1	11	0
			Normal (XX)	34	1	25	8
			Upper limit (>3.5)	1	0	0	1

**Table 5.** Point-of-care test results on venous blood samples compared to routine analyses – clinically relevant cut-offs.

			Measurements outside the clinically relevant cut-off				
Device	Biochemical parameter	Sample size	Laboratory		Point-of-care test venous blood samples		
					Lower limit	Normal	Upper limit
Hemocue 801	Hb, mmol/L	54	Lower limit (<7.3)	16	15	1	–
			Normal (≥7.3)	38	3	35	–
Hemocue WBF DIFF	Total WBC (10^9^/L)	50	Lower limit (<3.5)	0	0	0	0
			Normal (3.5–8.8)	22	0	21	1
			Upper limit (>8.8)	28	0	3	25
	Neutrophils (10^9^/L)	49	Lower limit (<2.0)	0	0	0	0
			Normal (2.0–8.8)	39	2	36	1
			Upper limit (>8.8)	10	0	3	7
	Lymphocytes (10^9^/L)	49	Lower limit (<1.0)	14	3	11	0
			Normal (1.0–3.5)	34	0	33	1
			Upper limit (>3.5)	1	0	0	1
iSTAT	Na^+^ (mmol/L)	47	Lower limit (<130)	2	2	0	–
			Normal (≥130)	45	0	45	–
	K^+^ (mmol/L)	47	Lower limit (<3.5)	9	6	3	0
			Normal (3.5–4.6)	33	0	31	2
			Upper limit (>4.6)	2	0	0	2
	Urea (mmol/L)	47	Normal (≤8.1)	31	–	26	5
			Upper limit (>8.1)	16	–	0	16
	Creatinine (µmol/L)	42	Normal (≤105)	31	–	28	3
			Upper limit (>105)	11	–	0	11

In venous blood testing, misclassification was observed when applying clinically relevant cut-offs: 4 out of 54 for haemoglobin, 4 out of 50 for total WBC, 6 out of 49 for neutrophils, 12 out of 49 for lymphocytes, 5 out of 47 for K^+^, 5 out of 47 for urea, and 3 out of 42 for creatinine. Misclassification did not occur for Na^+^ (Table 5).

## Discussion

Our results demonstrate a strong level of agreement between POCT measurements and routine laboratory measurements. However, clinicians should be aware that capillary blood samples often exhibit higher variation and reduced accuracy compared to venous blood samples. While this finding aligns with existing literature [5, 14–19], to our knowledge, this study is among the first to validate POCT in a cohort of older adults with signs of acute conditions during in-home assessments.

Among the analytes measured, lymphocytes exhibited the highest degree of misclassification, with venous POCT testing reducing the percentage of misclassification from 43% to 24%. Similar findings were reported by Kur et al. who noted a poorer correlation coefficient for lymphocytes in venous samples [5], though they did not compare capillary with venous samples. A paediatric study also highlighted a weaker correlation for capillary samples, suggesting that POCT for leukocytes using the Hemocue WBD DIFF should be limited to total leukocytes and neutrophil counts, rather than a full five-part differential count [14]. For other key measures such as Na^+^, K^+^, urea, creatinine, haemoglobin, leukocytes, and neutrophils, venous samples in our study demonstrated an acceptable degree of misclassification, indicating reliability for clinical use [12, 17, 18, 20, 21].

While most POCT can analyse both venous and capillary blood samples, capillary blood sampling proves particularly valuable in scenarios, where obtaining venous access is challenging. However, the limited volume will often restrict the range of accessible diagnostic parameters, and results may be influenced by variations in capillary blood flow and composition [22–24]. Furthermore, it is important to employ the correct capillary blood sampling technique for accurate results in haemoglobin and WBC differential counts. Factors such as cold fingers and poor blood circulation may pose challenges in obtaining an acceptable sample, potentially leading to inaccurate values. This often leads to a more brute manipulation in order to obtain the capillary sample, which could lead to a haemolysed sample.

POCT performed on venous blood samples, despite facing some pre-analytical challenges, showed stronger correlation with laboratory tests. Technical issues, specifically difficulty in locating veins, were the primary reasons for not collecting venous blood samples. Venous blood collection can be difficult to perform, especially in individuals with conditions such as dehydration, or those with veins that are fragile or difficult to visualize. In such cases, there are various strategies to enhance vein identification and improve the likelihood of successful blood collection. Techniques such as warming the sampling area or having the patient perform movements that raise blood flow may promote vein dilation. Utilizing ultrasound technology is another advanced method for visualizing veins, particularly in challenging cases, allowing for more precise identification and reducing the risk of multiple attempts [25]. Although our findings indicate that venous samples are better than capillary, the latter can be used when vein identification is difficult, but the clinician must be aware of a higher variation and inaccuracy for test results obtained with capillary samples, e.g. especially concerning the reliability on lymphocytes.

The utilization of POCT for measuring CRP, electrolytes, and haemoglobin offers valuable insights for clinicians. However, prior studies on the performance of POCT in older acutely ill cohorts are lacking. This study primarily aimed to validate POCT results against laboratory results, nested within a pilot and feasibility study [7]. The pilot and feasibility study explored the potential clinical impact of POCT on clinical decision-making, revealing that extended POCT during in-home assessment of older adults identified an additional 34% of participants with acute conditions requiring clinical decision-making by a physician. Another study in an out-of-hours outpatient emergency medical service demonstrated that the use of a CRP-POCT on capillary samples changed the clinical decision by physicians in 83.3% of cases. Notably, in 60% of all cases utilization of a CRP-POCT altered the decision regarding hospitalization, resulting in non-hospitalisation [26]. These results suggest that POCT can influence clinical decision-making, altering diagnoses and patient management. However, before implementing POCT in clinical settings, it is imperative to validate the equipment against routine laboratory results. Using POCT introduces greater uncertainty compared to laboratory testing, necessitating a basic knowledge on correlations, coefficient of variation (CV) values, and result variability. These factors are important for informed decision-making in diagnostics and treatment initiations, although they can pose challenges for clinicians. In our study, we evaluated the agreement between POCT and laboratory testing using scatter plots with linear regression, Pearson’s correlation coefficient (PCC), limits of agreement, and Bland-Altman plots. Additionally, we assessed the clinical reliability of POCT, by integrating clinically relevant cut-off points to evaluate misclassification rates, inspired by others [11].

When evaluating POCT, it is important to consider not only the correlation and variability relative to routine laboratory testing; it also entails considering clinically significant cut-off points. This approach ensures that clinicians can rely on the accuracy of test results, especially when values fall outside these critical thresholds.

In our study, we selected clinical cut-offs based on the laboratory’s normal reference intervals and in one case a clinically meaningful classifications. A designated cut-off for hyponatremia was set at less than 130 mmol/L, as higher measurements within the range of 130–135 mmol/L are often considered as mild and asymptomatic hyponatremia [12]. Similar considerations could apply for leukocytes, haemoglobin, and creatinine, although there is a lack of internationally agree-upon clinical cut-offs and sufficient studies for these measures [27–29].

The geriatric population, characterized by multi-morbidity, presents challenges in establishing clinically relevant cut-offs due to their heterogeneity [30–32]. Individuals may have known conditions like slightly elevated creatinine levels or anaemia, making it important to tailor and personalise treatment plans. Thus, while it is important to establish and investigate clinically relevant cut-offs, doing so in this specific population involves considerable difficulties, underscoring the need for individualised approaches and further research.

The potential implementation of POCT extends to various settings, including home care, doctor’s offices, and other healthcare facilities. Considering that infections and dehydration are common causes of emergency admissions among older adults [8], the inclusion of a comprehensive ‘analysis package’, encompassing white blood cell differential count, haemoglobin, creatinine, Na^+^, K^+^, and urea, holds promise for improving assessments and clinical decision-making in acutely ill older adults.

To address these challenges, conducting a comprehensive health technology assessment is advisable before fully implementing POCT [33–35]. Such an assessment would evaluate clinical impact, diagnostic accuracy, user perceptions, organizational implications, and cost-effectiveness, providing a balanced view of its feasibility and sustainability in healthcare settings.

The high rate of missing data affects our confidence in our study results (ranging from 26–42%). Initially, electronic transmission issues led to the first samples not being recorded in the system. We believe these data are missing at random and do not introduce a systematic bias into our estimates, although they do reduce our study’s power and hence our confidence in the results due to the diminished sample size. Additionally, missing data were also due to difficulties in obtaining venous access, which might reflect factors like the severity of illness or dehydration among participants, potentially affecting and introducing bias in our findings.

Our study sample consisted of older, multi-morbid patients, predominantly frail and dependent on home care – a population targeted due to their high rates of consultations with primary care physicians and frequent hospital admissions [31, 32]. The use of broad inclusion criteria and minimal exclusion criteria enhances the generalizability of our findings to the primary care setting. However, we acknowledge that excluding participants with moderate to severe cognitive impairment may limit the acceptability of our results to the broader geriatric population facing cognitive challenges.

Our study provides new insights into the validation of POCT versus routine laboratory testing in a cohort of older adults with signs of acute conditions – a group where its application is particularly promising but insufficiently studied. Given the small sample size, further research is needed to establish POCT’s clinical reliability in this demographic fully. We advocate for larger-scale studies to confirm our results and explore POCTs clinical impact further.

## Conclusion

In conclusion, our study findings suggest that POCT for haemoglobin, white blood cell differential count, creatinine, Na^+^, K^+^, and urea offer clinically reliable measurements in acutely ill older adults. However, it is important to approach these results with a critical perspective, acknowledging the inherent variations and uncertainties associated with blood sampling techniques and POCT analysis. Moreover, employing clinically relevant cut-offs, tailored to the specific setting and patient population, may enhance the clinical interpretation of results. We emphasize the importance of integrating clinical assessments with the POCT results to ensure comprehensive patient care. Continued research and refinement in POCT methodologies are important for advancing diagnostics and improving patient outcomes in acutely ill older adults.

In a clinical context, venous blood samples are preferable; however, capillary blood samples are acceptable for use during in-home assessments of acutely ill older adults, but clinicians must be aware of a higher variation and inaccuracy for test results obtained with these samples.
